# Factors enabling comprehensive maternal health services in the benefits package of emerging financing schemes: A cross-sectional analysis from 1990 to 2014

**DOI:** 10.1371/journal.pone.0201398

**Published:** 2018-09-25

**Authors:** Veronica Vargas, Sayem Ahmed, Alayne M. Adams

**Affiliations:** 1 Department of Economics, Universidad Alberto Hurtado, Santiago Chile; 2 David Rockefeller Center for Latin American Studies, Harvard University, Boston, Massachusetts, United States of America; 3 Health Economics and Financing Research Group, Universal Health Coverage, Health Systems and Population Studies Division, icddr,b, Dhaka, Bangladesh; 4 Health Economics and Policy Research Group, Department of Learning, Informatics, Management and Ethics, Karolinska Institutet, Stockholm, Sweden; 5 Department of International Health, Georgetown University, Washington, D.C., United States of America; 6 James P. Grant School of Public Health, BRAC University, Dhaka, Bangladesh; University Complutense of Madrid, SPAIN

## Abstract

**Introduction:**

Maternal delivery is the costliest event during pregnancy, especially if a complicated delivery occurs that requires emergency hospital services. A health financing scheme or program that covers comprehensive maternal services, including specialized hospital services in the benefits health package, enhances maternal survival and improves financial risk protection.

**Objectives:**

The objective of this study is to identify factors that enable the inclusion of comprehensive maternal services in the benefits package of emerging health financing schemes in low and middle-income countries across selected world regions. Comprehensive care is presumed if, in addition to normal delivery, primary health care, and secondary or tertiary hospital care are included.

**Methods:**

Multilevel regression analysis is performed on 220 health financing schemes and programs initiated during the period 1990–2014, in 40 countries in Sub-Saharan Africa, Asia, and Latin America.

**Findings:**

About two-thirds of emerging health financing schemes explicitly include maternal care in the benefits package, and less-than-half cover comprehensive maternal services. Provision of any type of maternal services and comprehensive services is significantly associated with the presence of donors/philanthropies as funders, and beneficiaries possessing an ID card that links them to entitled services. Other enabling factors are prepayment and risk pooling. However, private and community insurances are negatively associated with covering comprehensive maternal services, because they are subject to market failures, such as adverse and risk selection.

**Conclusions:**

Emerging health financing schemes in low and upper-middle-income countries lag in coverage of maternal care. Advancing financial protection of these services in the health package needs policy attention, including government oversight and mandatory regulations. The enabling factors identified can enrich the ongoing discourse on Universal Health Coverage.

## Introduction

The cost of healthcare has been identified as a major barrier facing women when they require maternal delivery services. Delivery is one of the single costliest events during pregnancy, especially for complicated deliveries requiring C-section which cost 2 to 13 times greater than normal facility deliveries [1]. Studies from Asia and Sub-Sahara Africa (SSA) estimate that maternal health care accounts for 1 to 5 percent of total annual household expenditure and can increase 5 to 34 percent if complications occur [2]. Moreover, paying for these services out-of-pocket or via user-fees is associated with an increased risk of financial hardship, and may result in affected households being pushed into poverty [3]. In low-income countries, estimates suggest that about 60 percent of total health expenditure (THE) including maternal services are out-of-pocket (OOP), while in middle-income countries OOP accounts for about one-third of THE [4]. Risk pooling mechanisms such as tax-financed national health service, mandatory social health insurance (SHI), voluntary private insurance (VPI) for-profit, or community health insurance (CHI) and prepayments, are designed to smooth the costs associated with seeking care. Enabling more equitable access to needed services than out-of-pocket payments [5–7], financial approaches involving risk pooling are widely favored in efforts to achieve affordable universal health coverage (UHC) [8].

Financial risk protection for maternity services is of particular importance, given the potentially life-threatening implications for women and children if time-sensitive emergency care is not obtained. Further, while basic delivery care including antenatal care services is anticipated for all pregnant women, the need for care associated with complications is uncertain. The fundamental objective of a financing scheme for healthcare is to minimize risk in the context of unpredictable health events by reducing the financial barriers that people face at the point of delivery [5, 6]. In this context, protection against the financial risks of unpredictable maternity-related emergencies is a critical policy concern.

This paper analyzes health financing schemes or programs emerging during the period 1990 to 2014 in LMICs in SSA, Asia, and Latin America, with a view to identifying factors that favor inclusion of comprehensive maternal services, in the benefits health package (BHP). Comparisons between schemes that do and do not provide maternal care in their BHP are performed, and if covered, factors associated with offering comprehensive maternal services are identified.

## Conceptual framework

**Fig 1:**
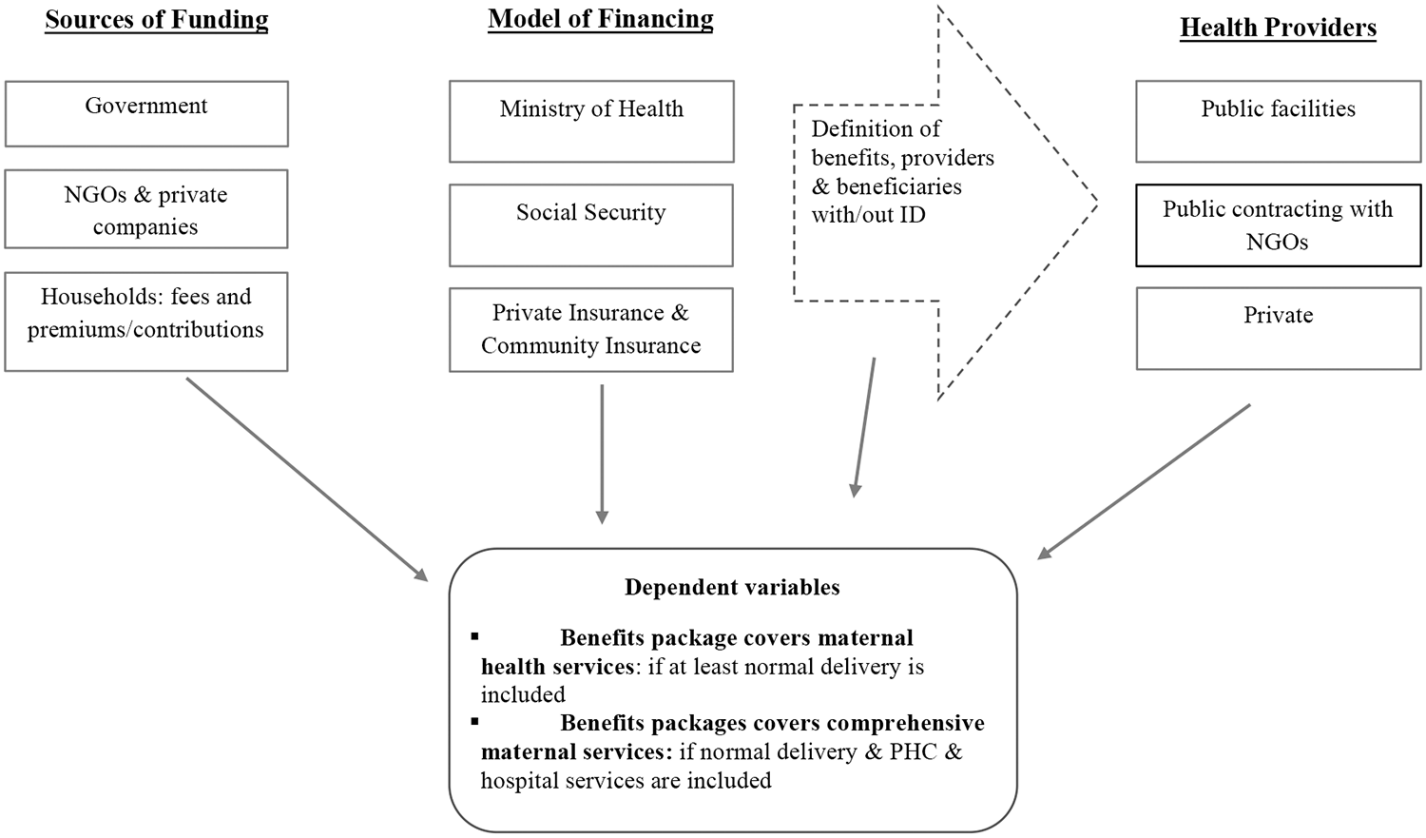
Relationships between health financing schemes attributes and coverage of maternal services in the BHP.

As shown in Fig 1, this paper seeks to identify which design attributes of health financing schemes are associated with the presence or absence of maternal services in the BHP of emerging health financing schemes. These design attributes include i) sources of funding, how the health financing scheme or program raises revenue–through taxes, mandatory contributions, voluntary premiums, fees, bi-multilateral organizations, and philanthropies, ii) model of financing, or the institutional arrangement for pooling the funding, iii) how beneficiary access to services is enabled, which generally entails the definition of a package of benefits and the provision of an individual/household identification (ID) card; and iv) health providers, or whether services are delivered in solo public facilities, public contracting with NGOs and private or NGOs facilities. Finally, the dependent variables, v) whether maternal care is provided at primary and/or secondary/tertiary levels.

### Benefit health package, funding sources and financing models

In the context of most financing schemes, the services made available can be left undefined, determined in general terms or made explicit. An explicit statement of the specific services to be financed sets clear entitlements for beneficiaries [9]. The maternal benefit package recommended by the World Health Organization (WHO), includes primary health care (PHC) and secondary level services. At the PHC level, pre and post-natal care, and skilled attendance at delivery should be provided by health professionals, while at the hospital level, skilled capacity for emergency obstetrics, including safe C-section should be available [10, 11]. Evidence suggests, however, that the required integration of primary and secondary level for comprehensive maternal services, which require cooperation and coordination, is often rare in poor-resource settings [12].

Regarding the sources of funding health, in LMICs, about 52 percent originates from public sources that include multilateral borrowing and grants, 36 percent from out-of-pocket and the remaining from other private sources [13, 4]. Because public funds to finance health have stagnated, loans from multilateral and grants from donors and philanthropies are particularly important in these countries [14]. Donor ‘support to maternal health, newborn and child health grew substantially during the 1990–2014 period, especially after 2000 [15].

In LMICs, the coverage of maternal services has been a priority for public programs delivered by Ministries of Health (MOH). Consequently, most LMICs are moving towards high coverage of essential maternal-health services [16]. But, access to maternal emergency such as C-section remains low, and among the poorest section of the population, it is estimated at 1–2 percent compared to 5–15 percent which is optimal for life-saving [17]. Some promising examples of improvements in emergency coverage include China’s rural New Medical Social Security scheme, where membership has been linked with reduced out-of-pocket expenditure for C-section, although not for normal delivery [18]. Another positive example is *Plan Nacer* in Argentina, where extended coverage of comprehensive maternal and child health services is targeting mainly low-income women [19].

The evidence on financial protection of maternal care by private insurance, both CHI and for-profit, is mixed. From the insurance perspective, normal delivery is a high-frequency, medium-cost event, while emergency delivery is a low-frequency, high-cost event. Thus, emergency obstetrics is considered an insurable risk, whereas normal delivery is regarded as “non-insurable,” and thus, most suitable for prepayment and public subsidies [7]. Some studies in SSA and Asia suggest that CHI members experience better financial protection from health shocks than do non-members [20,21]. However, private insurance experience from middle-high- income-countries shows that many of these schemes are subject to market failures due to adverse selection and risk selection [22, 23]. Consequently, CHIs often exclude services such as normal delivery [24]. Thus, while CHI membership may be positively associated with access to maternal care, it is the inclusion of maternal services in the BHP that makes the difference in providing financial protection.

Other financial instruments that help minimize out-of-pocket expenses are savings and prepayment schemes. With savings accounts, the financial risk of health expenditures can be spread over time [25]. For example, the “Birth-preparedness” program active in SSA, assists women in savings for anticipated child-birth expenses [26]. The sale of voucher booklets, a type of prepayment supplied by private outlets in Pakistan, has also been positively associated with increasing antenatal care and institutional delivery [27].

### Provision of services

Regarding the provision of maternal services, most LMICs rely on both public and private providers. Analysis of representative samples of maternal health providers in selected countries in SSA found that more than half of hospitals were private and three-quarters of PHC facilities were public [28]. In general, public systems function through a network of public providers, while in parallel SHI operates their own facilities. However, over the past decades, public programs have moved towards greater contracting out clinical and non-clinical services to private providers, including NGOs and faith-based organizations [29–30]. Voucher programs represent one modality of contracting out NGO providers, and stimulating demand by directly connecting services to intended beneficiaries [31]. Studies of government and donor-funded maternal voucher programs in Bangladesh and Cambodia have reported improved facility-based deliveries [32,33].

### Registration and identification

A final literature relevant to this analysis of enablers of comprehensive maternal care is the identification of beneficiaries. In many health insurance schemes, ID cards are issued following registration [34]. In Social Security Institutions, ID numbers are used to link an enrollee’s personal information to a defined set of entitlements, thus enabling access to services by contributing members, and extending coverage to non-contributing members [35]. Development banks involved in financing access to social programs highlight the importance of registration and IDs for beneficiaries in improving accountability [36,37,38]. In Thailand, the launch of insurance for UHC led to the registration and issue of a unique ID number, which is used to access services and for monitoring purposes [39]. Unique ID information facilitates data sharing across different levels of the health system, within the health financing scheme, and between primary and secondary level providers, thus improving patient continuity of care.

## Methodology

### The model

Multilevel logistic regression was used to predict the association between different design characteristics of the emerging health financing schemes and the coverage of maternal care in their benefit health package. We used this analysis to account for the hierarchical structure of the two levels of data. The primary level of the analysis is the financing scheme and program level, which is located and associated with the country level.

The models are represented by the following equations:

$$\text{Model}\;1\;logit\;({BHP}_{ij})\;=\;{\beta X}_{ij}+{\gamma \omega }_{j}+r_{ij}$$ (1)

$$\text{Model}\;2\;logit\;({BHPM}_{ij})\;=\;{\beta X}_{ij}+{\gamma \omega }_{j}+r_{ij}$$ (2)

Two binary dependent variables were used. BHP*ij* is the benefits package of the health financing scheme *i* in country *j*. If normal delivery is included, BHP equals 1 otherwise 0. Whereas, BHPM*ij* represents the package covering comprehensive maternal services, equaled to 1 otherwise 0. We assume a BHP includes normal delivery if maternal care is covered. Comprehensive maternal care is presumed if, in addition to maternal care, PHC and secondary or tertiary hospital care are explicitly stated as part of the BHPM. Finally, we assume that PHC included antenatal and post-natal care, and that secondary/tertiary hospital level care includes emergency care (S1 Appendix).

Where, X*ij* is a vector of characteristics of *ith* health financing schemes located in *jth* country and *wj* is a set of observed and unobserved country characteristics. The coefficient *β* characterizes the association between individual health financing scheme characteristics (year of inception, sources of funding, financing model, health provider, possession of ID and the host country socioeconomic level) and the coverage or absence of maternal care in the benefits package whereas; γ the country variable, captures the unobserved variance among each country and the coverage or absence of maternal care in the benefits package. The r*ij* is the model intercept. We estimated the odds ratio and it’s 95% confidence interval from this analysis.

The variable year of inception of the health scheme *i*, is a continuous variable and ranges from the year 1990 to 2014. It was centered at 1990 (or equals zero), so as to produce interpretable baseline odds from the logistic regression models.

The sources of funding variable include three dummies: donors/philanthropy and bi-multilateral, prepayment or households’ contributions and premiums and households’ user-fees. If ´the financing scheme *i* received any funding from described sources, then the variable equals 1, otherwise it was 0.

The financing model variable was captured by two dummies: Ministry of Health plus Social Health Insurance, and Private and Community Health Insurance. If financing scheme *i* fits the dummy description then equals to 1, otherwise it was zero.

The health provider type is grouped into three dummies: only public provider, public sector contracts with NGOs and other private provider. If financing scheme *i* satisfies the described category then the dummy variable equals 1, otherwise 0.

The variable IDi is a dummy variable and is equated to 1 if the financing scheme *i* provides identification to members, otherwise it was equal to zero.

The variable country-income level, was constructed using three dummies, based on the categories defined by World Bank [4], low-income per-capita equals $1,045 or less, lower-middle-income per-capita between $1,046 to $4,125, and upper-middle-income per-capita between $4,126 to $12,735. If the home-country of the financing scheme *i* fell into the defined range in the year 2016, then it was equal to 1, otherwise it was 0.

Descriptive statistics, such as mean, frequency, and percentage, were estimated to analyze the 220 cases. The odds ratios (OR) resulting from the multiple logistic regression are reported with confidence intervals (95% CI) and p-values of 0.01 (three starts), 0.05 (two starts) and 0.10 (one start).

### Data

Secondary data were drawn from a dataset compiled by Results for Development Institute that captures all more than 1500 known health insurance schemes and programs for uncovered populations, in 130 LMIC countries [40], and provides a one-page description of each program/scheme serving the poor and near-poor. After the filter “health focus” and “financing care” were applied, the number of cases was reduced to 410. Additional exclusion criteria removed: i) schemes that covered only the formal working population, ii) schemes that included only one health intervention, for example, family planning, or dentistry, iii) schemes that no longer existed, iv) schemes located outside Asia, SSA or Latin America, and iv) schemes starting before 1990. This produced a final dataset of 220 schemes. Missing information from this dataset was complemented with a second set “Compendium of Microinsurances” gathered by the International Labour Organization [41] and by visiting the web page of each scheme, if available. Also, searches were performed in Google and Google Scholar using the keywords: name of the scheme, maternal delivery services, country, and ID card (S2 Appendix).

## Results

### Descriptive features of emerging health financing schemes and programs

**Fig 2:**
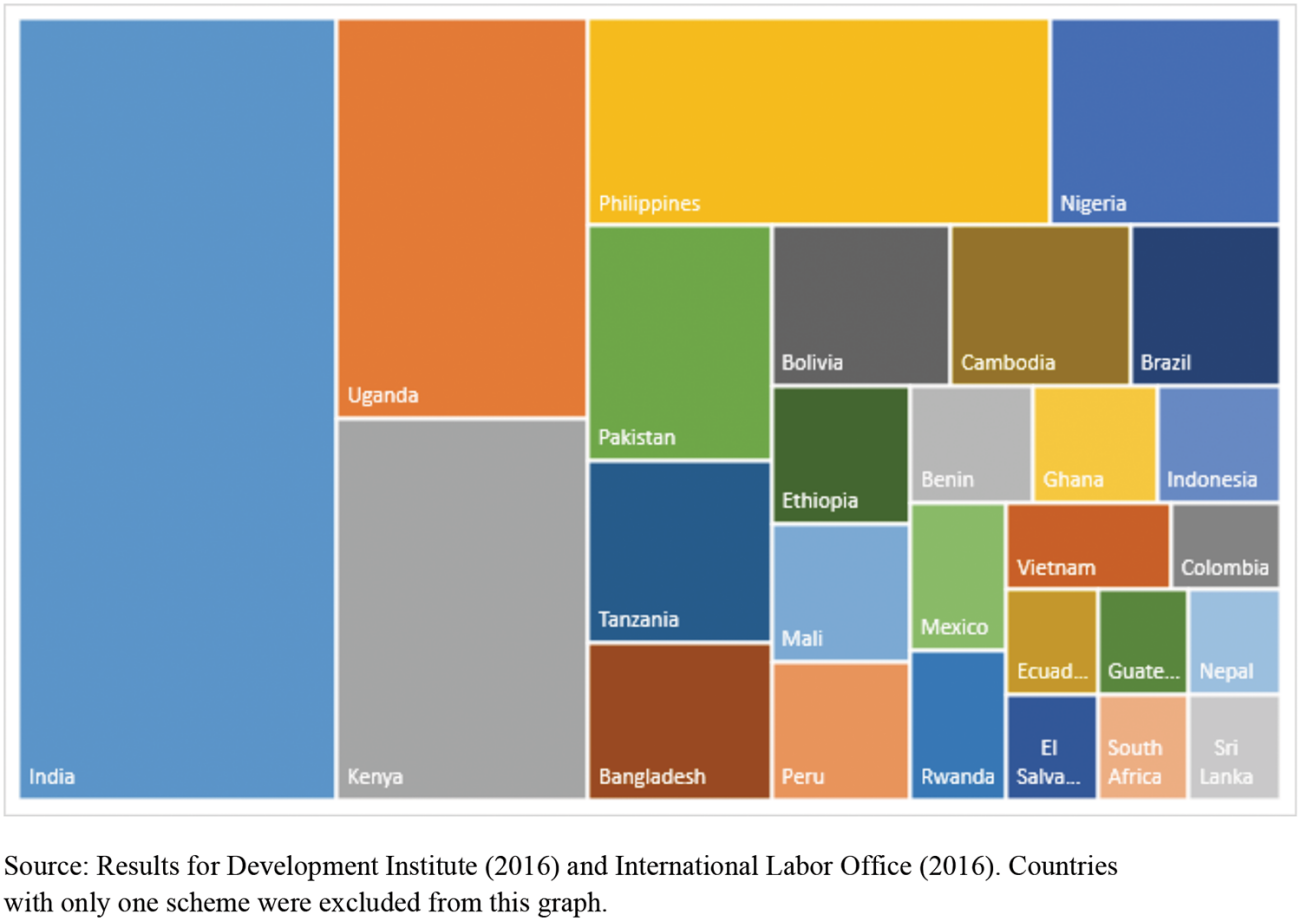
Countries from Sub-Saharan Africa, Asia and Latin America with emerging health financing schemes from 1990–2014.

This analysis includes 220 financial schemes and programs established between 1990 and 2014 in 40 countries across SSA, Asia, and Latin America. The average year of initiation was 2004, with a mean duration of 11 years, in 2015. Twelve countries hosting emerging health financing schemes belong to a lower-income group, eighteen falls in the lower-middle-income category and ten into the upper-middle-income group. About 60 percent of the schemes are concentrated in six countries: India, Uganda, Kenya, Philippines, Nigeria, and Pakistan (Fig 2).

Out of the 220 schemes, 66 percent guarantee access to maternal services, and 44 percent provide comprehensive care services, which includes normal delivery, services at PHC and hospital level. As shown in Table 1, when health financing schemes are classified based on their host country economic level the large majority (65 percent) are located in lower-middle income countries, and 25 percent in low-income countries. Upper-middle-income countries host only one-tenth of schemes.

**Table 1:** The design characteristics of emerging health financing schemes/programs according to whether or not they provide maternity services in their benefits health package according to two model specifications.

Variables	Total universe of schemes/programs	Model 1 BHP covering any maternal services		Model 2 BHP covering comprehensive maternal services	
		Yes	No	Yes	No
**Benefits health package: level of care**					
Only primary health care	45 (100.0%)	21(46.7%)	24 (53.3%)	0 (0%)	45(100,0%)
Only secondary or tertiary hospital	47 (100.0%)	29 (61.7%)	18 (38.3%)	0 (0%)	47(100,0%)
Primary health care & hospitals	128(100.0%)	96 (75.0%)	32 (25.0%)	96 (75.0%)	32(25,0%)
**Total**	220 (100.0%)	146 (66.4%)	74 (33.6%)	96 (43.6%)	124(56,4%)
**Income level of home country**					
Low-income^1^	54 (100.0%)	41 (75.9%)	13 (24.1%)	28 (51.9%)	26(48,1%)
Lower-middle-income^2^	143 (100.0%)	96 (67.1%)	47 (32.9%)	62 (43.4%)	81(56,6%)
Upper-middle-income^3^	23 (100.0%)	9 (39.1%)	14 (60.9%)	6 (26.1%)	17(73,9%)
**Total**	220 (100.0%)	146 (66.4%)	74 (33.6%)	96 (43.6%)	124(56,4%)
**Funding from households**					
User-fees	29 (100.0%)	15 (51.7%)	14 (48.3%)	6 (20.7%)	23(79,3%)
Prepayment: contributions and premiums	150 (100.0%)	102 (68.0%)	48 (32.0%)	73 (48.7%)	77(51,3%)
Other non-households	41 (100.0%)	29 (70.7%)	12 (29.3%)	17 (41.5%)	24(58,5%)
**Total**	220 (100.0%)	146 (66.4%)	74 (33.6%)	96 (43.6%)	124(56,4%)
**Funding from donors**					
Donors/philanthropies & bi-multilaterals	103 (100.0%)	74 (71.8%)	29 (28.2%)	49 (47.6%)	54(52,4%)
Non-donors	117 (100.0%)	72 (61.5%)	45 (38.5%)	47 (40.2%)	70(59,8%)
**Total**	220 (100.0%)	146 (66.4%)	74 (33.6%)	96 (43.6%)	124(56,4%)
**Main financing models**					
Ministry of Health or Social Health Insurance	81 (100.0%)	61 (75.3%)	20 (24.7%)	44 (54.3%)	37(45,7%)
Private Health Insurance or Community Health Insurance	119 (100.0%)	72 (60.5%)	47 (39.5%)	45 (37.8%)	74(62,2%)
Other	20 (100.0%)	13 (65.0%)	7 (35.0%)	7 (35.0%)	13(65,0%)
**Total**	220 (100.0%)	146 (66.4%)	74 (33.6%)	96 (43.6%)	124(56,4%)
**Providers**					
Only public providers	31(100.0%)	21(67.7%)	10(32.3%)	16(51.6%)	15(48,4%)
Public sector contracting NGOs or private providers	74(100.0%)	57(77.0%)	17(23.0%)	37(50.0%)	37(50,0%)
Other private providers	115(100.0%)	68(59.1%)	47(40.9%)	43(37.4%)	72(62,6%)
Total	220(100.0%)	146(66.4%)	74(33.6%)	96(43.6%)	124(56,4%)
**Identification**					
Without ID card	40 (100.0%)	23 (57.5%)	17 (42.5%)	12 (30.0%)	28(70,0%)
With ID card	180 (100.0%)	123 (68.3%)	57 (31.7%)	84 (46.7%)	96(53,3%)
Total	220 (100.0%)	146 (66.4%)	74 (33.6%)	96 (43.6%)	124(56,4%)

As defined by World Bank (2016):

^1^Afghanistan, Benin, Cambodia, Ethiopia, Guinea, Liberia, Malawi, Mali, Nepal, Rwanda, Tanzania, Uganda,

^2^Bangladesh, Bolivia, Cameroon, El Salvador, Ghana, Guatemala, India, Indonesia, Kenya, Lao PDR, Lesotho, Nicaragua, Nigeria, Pakistan, Philippines, Sri Lanka, Vietnam, Zambia.

^3^Botswana, Brazil, China, Colombia, Ecuador, Mexico, Peru, South Africa, Thailand.

**Fig 3:**
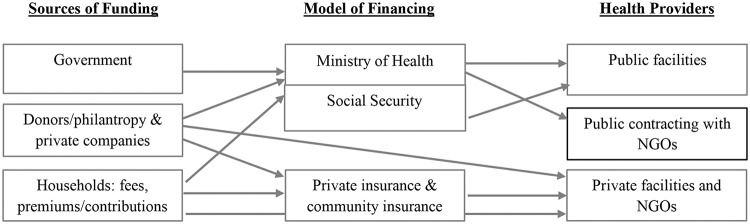
Funding sources and flow of funds to financial institutions and health providers.

Funding for schemes and programs that include maternal care in BHP comes from multiple sources (Fig 3). Around 80 percent of schemes rely wholly or partially on households funding via premiums, contributions and user-fees. At the same time bi-multilateral, donors and philanthropies contribute with grants and loans to almost one-half, including public programs, community insurance, NGOs providers, and other private providers. Finally, 37 percent of programs rely on public funding (Table 1). With respect to the model of financing, almost 90 percent of schemes in this review pool the funds, if we combine Ministries of Health, Social Security and both types of private insurances. Within the cluster of schemes offering comprehensive maternal care, this proportion increases to 93 percent.

As shown in Table 1, also displays the financing arrangement of emerging financial schemes is shared between private and community health insurance funded with premiums paid by households. This is followed by the tax-funded government and social health insurance programs, with the remaining schemes relying on user-fees or co-payments (other).

Each scheme in the dataset, is classified into one of the three following categories: solo public delivery, public sector programs contracting with NGOs or other private provider for delivery of services, and a remaining category that includes mostly solo private providers or NGOs (Table 1). In the total universe of schemes and programs included in this analysis, public programs, solo or contracting with NGOs or private providers, represent 48 percent of overall providers, with solo providers making up the remainder. In contrast, under Model 1 which represents the cluster of schemes providing maternal services, the proportion of public providers, solo or contracting with NGOs, increases to 53 percent of total schemes while the share of private providers decreases to 47 percent.

Regarding the possession of ID variable, the overall proportion of schemes involving registration with personal ID health cards extends to 82 percent of schemes and programs analyzed. In schemes with an ID card, 47 percent offer comprehensive maternal care, whereas, among those without ID card, only 30 percent offer the same.

### Effects of health financing scheme attributes on coverage of maternal services

Table 2 presents the results of multilevel logistic regressions analysis in terms of odds ratios (OR) and their corresponding confidence intervals. A hierarchical structure is used in order to detect the inter-level effects, with the health schemes and programs in level 1 and the country-context effects in level 2. In this analysis, the relative contributions of the various independent variables to the inclusion of maternal care in the schemes’ BHP are estimated, under two model specifications. The first model examines the presence of any type maternal services in the BHP, while the second considers only those schemes that provide comprehensive maternal services in the scheme’s BHP.

**Table 2:** Coverage of maternal delivery services in the benefits health package of emerging financing schemes by design attributes: Multilevel logistic regression, odds ratios 95% CI. Reference category equals 1.000.

	Model 1 BHP covering any maternal services	Model 2 BHP covering comprehensive maternal services
**Income level of home country**		
Low-income^1^	1.000	1.000
Lower-middle-income^2^	0.807 [0.366–1.779]	0.793 [0.392–1.603]
Upper-middle-income^3^	0.259** [0.078–0.856]	0.434 [0.130–1.449]
**Financing schemes attributes**		
**Year of inception**	0.951* [0.903–1.002]	1.000 [0.953–1.051]
**Funding from households**		
User-fees	1.000	1.000
Prepayment; contributions and premiums	1.574 [0.583–4.252]	3.607** [1.235–10.534]
Other non-households	1.778 [0.546–5.791]	2.363 [0.685–8.149]
**Funding from donors**		
Non-donors	1.000	1.000
Donors/philanthropies & bi-multilaterals	2.228 ** [1.081–4.593]	2.436** [1.231–4.823]
**Main financing models**		
Ministry of Health or Social Health Insurance	1.000	1.000
Private insurance or Community Health Insurance	0.379* [0.131–1.096]	0.235*** [0.091–0.608]
Other	0.686 [0.168–2.801]	0.367 [0.099–1.358]
**Providers**		
Only public providers	1.000	1.000
Public sector contracting NGOs or private providers	1.925 [0.661–5.609]	0.925 [0.355–2.407]
Other private providers	0.827 [0.285–2.401]	0.903 [0.326–2.498]
**Identification**		
Without ID card	1.000	1.000
With ID card	3.554** [1.111–11.376]	2.579* [0.897–7.416]
**Constant**	1.444 [0.234–8.898]	0.254 [0.042–1.539]
**Country random effect**	0.000	0.000
**N schemes/programs**	218	218
**N country groups**	40	40
**Prob > chi2**	0.007***	0.009***
**Log likelihood**	-123.42992	-133.71976

P value < 0.01 equals ***, P value < 0.05 equals ** and P value < 0.10 equals *

^1^ Low-income GDP per capita $1,045 or less: Afghanistan, Benin, Cambodia, Ethiopia, Guinea, Liberia, Malawi, Mali, Nepal, Rwanda, Tanzania, Uganda.

^2^Lower-middle-income GDP per capita $1,046 to $4,125: Bangladesh, Bolivia, Cameroon, El Salvador, Ghana, Guatemala, India, Indonesia, Kenya, Lao PDR, Lesotho, Nicaragua, Nigeria, Pakistan, Philippines, Sri Lanka, Vietnam, Zambia.

^3^Upper-middle-income GDP per capita $4,126 to $12,735: Botswana, Brazil, China, Colombia, Ecuador, Mexico, Peru, South Africa, Thailand.

Multicollinearity between independent variables is assessed using the variance inflation factor (VIF), since the independent variables belong to a conceptually intertwined health financing framework (Figs 1 and 3). As a result, the “only public funding” category under the variable “sources of funding”, has been dropped from the regression analysis. The resulting VIF, after excluding only public funding and the country variable, was 2.08 (1.02–3.56), which is an acceptable value.

Since the schemes and programs in this analysis are found in 40 different countries, we controlled for differences in outcomes that might be related to unobserved country-level characteristics related to demographic structure, socio-economic institutions, health infrastructure, or legislation on maternal care provision. To evaluate, if a fixed or random effects model was more appropriate for controlling for country effects, a Hausman test was performed. The Hausman test was not significant (p > 0.05), and therefore, a random effects model was used, by means of a multilevel mixed effect, using the command melogit in Stata version 15. The results show that there is nonsignificant variance, which indicates that there would be no difference across countries in the probability of health schemes and programs to include maternal care in their benefit package. It should be noted that a classical logistic regression analysis was also conducted, without nesting, and the results obtained were very similar to the nesting model.

The following independent variables were used: economic level of the country, year of scheme inception, sources of funding, health financing model, health provider arrangement, and possession of ID card.

Across both models, design attributes that significantly predict the likelihood of including maternal services in the BHP are donors/philanthropist as a source of funding, the private insurance as model and possession of ID. Type of provider had no effect is either model specification. Some variables were significant in just one model, such as country income level and year of inception in Model 1, and household as source of funding in Model 2.

In Model 1, the presence of any type of maternal care services in the BHP, is positively and significantly associated with donors/philanthropies and bi-multilateral as a source of funding. In other words, funding from these sources doubles the likelihood that any type of maternal care is offered in the BHP compared to funding from other sources. Similarly, beneficiary registration and the issuance of ID-card, increase the likelihood of offering maternal care in the BHP between 2.6 to 3.5 times in comparison with schemes not issuing IDs. In contrast, regarding the financing model, results indicate that the presence of private or community health insurance is negatively associated with the provision of maternal care including comprehensive maternal services, in comparison to public institutions such as the MOH and SHI.

Regarding the coverage of comprehensive maternal care (Model 2), this is positively and significantly associated with prepayment or households contributing or paying premiums to Social Health Insurance institutions or private insurance, in comparison to relying on paying user-fees to providers.

With respect to country income level in Model 1, the odds of providing maternal care decreases considerably for schemes located in upper-middle-income countries—largely private insurance schemes—compared to those in the low-income group. The year at which the scheme was initiated also shows some impact on the presence of maternal care, with schemes initiated earlier significantly more likely to include maternal care in their benefits package (Table 2).

Finally, both models come out highly significant when statistically validated using the Chi-square test. In summary, multilevel logistic regression analysis shows that the factors with the most significant and positive association with both the coverage of any type of maternal care and comprehensive maternal care in the BHP, are the presence of donors/philanthropy and bi-multilaterals as funders, and the identification of beneficiaries. Also, public financing emerged as a positive force, in comparison to private and community insurance.

## Discussion

Results from this study enrich the ongoing discourse on UHC by providing insights on the design attributes of health financing schemes that enable financial protection for maternal health services. Findings suggest that schemes driven by the public sector and involving donors as sources of funding, are a positive force in ensuring that maternal services are included BHP. The importance of ID provision and health entitlements is also indicated. At the same time, the limits of public-private collaboration in increasing maternal coverage are revealed, suggesting the need for proper regulation from the government.

However, findings should be qualified, since our analysis does not assume that the schemes and programs included in the database represent the totality of health financing schemes emerging during the study period. While, Results for Development aims to census all such programs, the likelihood for inclusion was greater among those schemes and programs posted electronically or documented in the English language. As a consequence, regions such the Middle-East, Eastern Europe and some Latin American countries are underrepresented, and/or incompletely documented.

Another note of caution concerns the non-significant country effect, which implies that the probability to include maternal care in the benefits package does not vary by country, except for the differences captured by the country income level variable. An explanation may be that the inclusion of maternal services in the benefits package, of private and community health insurance schemes, belongs to the micro-level analysis, and is mostly independent of long term country-specific trends. An alternative explanation might be found in the skewness of country observations included in the analysis. Because only 27 out of 40 countries have more than one scheme the estimates summarizing country effects at level 2, may not be as reliable as schemes and programs estimates from level 1 [43]. On this point it should be noted, that a classical logistic regression was also conducted without nesting, and the results obtained were similar to the nesting model.

Regarding essential services, results show that about half of new emerging financial schemes cover hospital services and maternal care. This is especially apparent among private financing schemes, which play an increasing role in emerging health financing in LMICs. From a behavioral economics perspective, it is difficult for individuals and households to plan for uncertain low-probability events, such as maternal emergency care. Further, failing to prepare will have greater consequences for the poor [42, 3]. This lacuna merits government intervention and mandatory regulations. An example is Ghana, where maternal coverage has been made compulsory in the BHP of commercial and community health insurance schemes [44].

With respect to the role of donors and philanthropies, whether the principal source of funding or not, this analysis brings new evidence to the discussion of their contribution to maternal health. In this analysis, their presence increases the likelihood that maternal delivery and comprehensive maternal services are covered in the BHP. This may be due to the fact that many of these schemes emerged during the Millennium Development Goals implementation period (2000–2015). Under this framework, global goals and targets related to the reduction of maternal mortality may have contributed to increasing earmarked financial donor support directed to maternal programs [1–16].

Regarding comprehensive maternal services, findings confirm the consensus viewpoint that prepaid schemes increase financial protection, in comparison to user-fees [5, 7, 8]. However, some exceptions occur, as certain prepaid schemes, such as voluntary private and community health insurance, are less likely to offer maternal care, in comparison to Ministries of Health and Social Security Institutions. This can be understood in terms of adverse selection and risk-selection or “cherry-picking.” To protect against adverse selection, some voluntary private insurance schemes risk-select and may exclude maternity benefits from the benefits package or impose waiting periods of at least nine months, to prevent already pregnant women from enrolling. For example, our findings show that there is a gap by private and community health insurance in covering maternal care at the hospital level; 34 percent of the schemes that cover hospital care do not cover maternal care. This finding suggests a deliberate exclusion of maternal hospital services and the practice of risk-selection by some schemes. However, the majority of private schemes that cover hospital care, also cover maternal care, which suggests that overall the relationship between private and community health insurance and coverage of maternal care at the hospital level is positive. To further encourage the inclusion of maternal care, a variety of innovations hold promise such as bundling health insurance with other financial services, partnering with high-volume health providers, or using risk-adjusted payment methods [28, 23].

Finally, this study provides preliminary evidence that registered, identified and entitled members are critical factors in schemes that offer a BHP that includes coordination-intensive services such as comprehensive maternal care. However, this finding is based on scheme and program as the unit of analysis. Future research using individuals as the unit of analysis, is needed to confirm both the direction and strength of the relationship between ID and health entitlements. A robust system of identification enables the smooth delivery of services that require coordination within the financial scheme itself, and among various primary and secondary/tertiary level providers, and beneficiaries. It is also notable that many traditional public health programs in lower-income countries lack ID systems. Although this is changing, as exemplified by the Thai Health Card Program and the Bolivian Maternal and Child Health Insurance, a significant expansion may substantially improve the implementation of programs that are coordination-intensive.

## Conclusions

Over the last twenty-five years, more than two hundred new health financing schemes and programs have emerged in LMICs in SSA, Asia, and Latin America to serve uncovered populations. However, the BHP of these schemes lags in coverage of maternal health, especially comprehensive maternity services, which includes emergency hospital care, the need for which, is difficult-to-predict, plan and coordinate.

Health financing schemes that provide financial protection to uncovered populations and include both comprehensive and non-comprehensive maternal care in their BHP, are positively associated with the presence of donors/philanthropies as funders and the identification of entitled beneficiaries. In addition, prepayment and risk-pooling arrangements are associated with the inclusion of comprehensive maternal services in the BHP. However, pooling is not sufficient, equally important is the explicit inclusion of these benefits in the package, which in the case of private and community insurance, is vulnerable to market failures and requires government regulation and oversight.

Our findings provide new evidence that contributes to various literature streams related to the design of financing schemes and essential health services, such as comprehensive maternity coverage. The enabling conditions identified may also enrich ongoing discussions supporting progress towards UHC.

## Supporting information

S1 Appendix Codebook of variables. (DOCX)

S2 Appendix List of financing schemes in Sub Sahara Africa, Asia and Latin America 1990–2014. (DOCX)
