# Supplementary material for: Factors enabling comprehensive maternal health services in the benefits package of emerging financing schemes: A cross-sectional analysis from 1990 to 2014
Source: PLoS One. 2018 Sep 25;13(9):e0201398. doi: 10.1371/journal.pone.0201398 (PMC6155500; doi:10.1371/journal.pone.0201398)
Supplement: S1 Appendix — (DOCX) [file pone.0201398.s001.docx]

**S 1 Appendix. Codebook of variables**

| Scheme or program name |  |
| --- | --- |
| Year started |  |
| Duration |  |
| ID | Yes  Not |
| Country |  |
| Country income level | Low-income  Lower-middle-income  Upper-middle-income |
| Region | Africa  Asia  Latin America |
| Health services | General primary care  General secondary/tertiary care/emergency  Maternal delivery and child care |
| Sources of funding | Public funds  Membership/subscription fees  Donor, philanthropy, multilateral, bilateral  Out-of-pocket payments |
| Financing models | Government: Ministry of Health  Government: Social Health Insurance  Micro/community health insurance  Private health insurance  Other: health saving, voucher, loan |
| Provider | Ministry of Health/Social Security public  Public contracting private provider  NGOs – private  Private -for-profit |
