# Supplementary material for: Factors enabling comprehensive maternal health services in the benefits package of emerging financing schemes: A cross-sectional analysis from 1990 to 2014
Source: PLoS One. 2018 Sep 25;13(9):e0201398. doi: 10.1371/journal.pone.0201398 (PMC6155500; doi:10.1371/journal.pone.0201398)
Supplement: S2 Appendix — (DOCX) [file pone.0201398.s002.docx]

**S2 Appendix. List of financing schemes in Sub Sahara Africa, Asia and Latin America 1990-2014**

| **N** | **Name** | **Country** |
| --- | --- | --- |
| 1 | NGO Performance-Based Contracting | Afghanistan |
| 2 | Sajida Foundation Health Program | Bangladesh |
| 3 | Grameen Kalyan Health Program | Bangladesh |
| 4 | Bangladesh Second Urban Primary Health Care Project | Bangladesh |
| 5 | Bangladesh Urban Primary Health Care Services Delivery Project | Bangladesh |
| 6 | Bangladeshi Rural Advancement Committee (BRAC) | Bangladesh |
| 7 | Dhaka Urban Community Health Program | Bangladesh |
| 8 | Association d'Entraide des Femmes (AssEF) | Benin |
| 9 | Benin's National Health Insurance (RAMU) | Benin |
| 10 | L'Union Communale des Groupements Mutualistes | Benin |
| 11 | Sol Salud | Bolivia |
| 12 | Fundacion Cuerpo de Cristo (FCC) | Bolivia |
| 13 | Healthy CRECER | Bolivia |
| 14 | PROSALUD | Bolivia |
| 15 | CREDISALUD | Bolivia |
| 16 | Pro Mujer Bolivia | Bolivia |
| 17 | Itekanele Health Scheme | Botswana |
| 18 | Hospital Dr. Moyses Deutsch | Brazil |
| 19 | Clinicas Da Familia | Brazil |
| 20 | Programa Mãe Paulistana | Brazil |
| 21 | Hospital do Subúrbio | Brazil |
| 22 | Projeto CIES (Center of Education and Health Integration) | Brazil |
| 23 | Sihanouk Hospital Center of Hope Clinic | Cambodia |
| 24 | Community-Based Health Insurance | Cambodia |
| 25 | Cambodian Association for the Assistance to Families and Widows (CAAFW) Community Health Insurance | Cambodia |
| 26 | My Family's Health (MFH) | Cambodia |
| 27 | Sokapheap Krousat Yeugn (SKY) Micro-Health Insurance | Cambodia |
| 28 | Health Equity Fund Managed by Pagoda | Cambodia |
| 29 | Cameroon Health Sector Support Investment Project | Cameroon |
| 30 | China's Rural Mutual Health Care (RMHC) | China |
| 31 | General System of Social Security in Health -- the Subsidized Regime | Colombia |
| 32 | General System of Social Security in Health (Colombia) | Colombia |
| 33 | Fundación Futuro | Ecuador |
| 34 | Unión Católica de Apoyo al Desarollo Comunitario (UCADE) | Ecuador |
| 35 | Apoyo Integral | El Salvador |
| 36 | FIMRC Micro Health Insurance Program (MHIP) | El Salvador |
| 37 | Ethiopia Community-Based Health Insurance | Ethiopia |
| 38 | Ethiopia Social Health Insurance | Ethiopia |
| 39 | Saint Yared Health Maintenance Plan | Ethiopia |
| 40 | Organization for Women in Self Employment (WISE) | Ethiopia |
| 41 | Nkoranza Community Financing Health Insurance Scheme | Ghana |
| 42 | Be Alert | Ghana |
| 43 | Ghana National Health Insurance Scheme | Ghana |
| 44 | NGO Contracting in Guatemala | Guatemala |
| 45 | Aseguradora Rural Microinsurance | Guatemala |
| 46 | L'Union des Mutuelles de Sante de Guinee Forestiere (UMSGF) | Guinea |
| 47 | Awareness Swasthya Bima Yojana | India |
| 48 | Mukhya Mantri Jiban Jyoti Bima Asoni Insurance | India |
| 49 | Chief Minister's Comprehensive Health Insurance Scheme | India |
| 50 | Andhra Pradesh Urban Slum Health Care Project (APUSHCP) | India |
| 51 | Aysuhmathi scheme | India |
| 52 | Kalaignar Insurance Scheme | India |
| 53 | Rashtriya Swasthya Bima Yojana (RSBY) | India |
| 54 | Vajpayee Arogyasri | India |
| 55 | Arogya Rath: Mobile Medical Units (MMU) in Bihar | India |
| 56 | Contracting of selected district health care facilities in Uttar Pradesh | India |
| 57 | Deen Dayal Chalit Aspatal (Mobile Units) | India |
| 58 | PPP Super Specialty Hospitals in Punjab | India |
| 59 | Ayushmati Scheme | India |
| 60 | Janani Express Madhya Pradesh | India |
| 61 | Janani Express Orissa | India |
| 62 | Janani Sahayogi Yojana | India |
| 63 | CARE Hospitals | India |
| 64 | Alka Hospital : Sampurna Suraksha Card | India |
| 65 | Anjali Microfinance Health Insurance | India |
| 66 | BASIX Microinsurance | India |
| 67 | CARE India Microinsurance | India |
| 68 | Kathir Foundation | India |
| 69 | Mayapur Trust | India |
| 70 | NIDAN Microinsurance | India |
| 71 | SKDRDP Trust | India |
| 72 | Swashraya Health Card Programme | India |
| 73 | Swasthya Pratham | India |
| 74 | Vimo SEWA | India |
| 75 | Ensuring the Poor in Rural India | India |
| 76 | Hello Doctor 24x7 | India |
| 77 | Association for Health Welfare in the Nilgiris (Ashwini) | India |
| 78 | Yeshasvini Cooperative Farmers Health Care Scheme | India |
| 79 | Indira Kranti Patham (IKP) | India |
| 80 | Merrygold Health Network | India |
| 81 | Aarogyasri Community Health Insurance Scheme | India |
| 82 | Deepak Foundation Gujarat | India |
| 83 | Karra Society for Rural Action - Referral Networks in Jharkhand | India |
| 84 | NICE Foundation | India |
| 85 | RCH Guwahati | India |
| 86 | Arpana Swasthya Kendra | India |
| 87 | NYSASDRI, Management of Primary Health Centers | India |
| 88 | Arogya Raksha Yojana Health Micro Insurance | India |
| 89 | CARE Rural Health Mission | India |
| 90 | Sampoorna Suraksha (Micro Health Insurance) | India |
| 91 | SHEPHERD | India |
| 92 | Swayam Shikshan Prayog (SSP) | India |
| 93 | Uplift Health Mutual Fund (HMF) | India |
| 94 | Kalanjiam Foundation | India |
| 95 | Healing Fields Foundation-Micro Health Insurance | India |
| 96 | LifeSpring Hospitals Private Limited (LHPL) | India |
| 97 | Amrita Institute of Medical Science (AIMS) | India |
| 98 | Narayana Hrudayalaya Hospital (NH) | India |
| 99 | Jamkesmas Scheme | Indonesia |
| 100 | The Health Safety Net for Poor Families (JPK Gakin/SKTM) | Indonesia |
| 101 | TAMADERA Micro Health Insurance | Indonesia |
| 102 | Afya Milele Halisi | Kenya |
| 103 | Faulu | Kenya |
| 104 | Equity Bank | Kenya |
| 105 | Linda Jamii | Kenya |
| 106 | The Health and ROSCA Project (HARP) | Kenya |
| 107 | CIC M-BIMA | Kenya |
| 108 | Global MamaCare Initiative | Kenya |
| 109 | National Social Health Insurance Fund | Kenya |
| 110 | Jamii Smart | Kenya |
| 111 | Bima ya Jamii Project | Kenya |
| 112 | Changamka Microhealth Limited | Kenya |
| 113 | Jamii Bora Trust | Kenya |
| 114 | M-Afya Kiosks | Kenya |
| 115 | Mamakiba | Kenya |
| 116 | Co-operative Insurance Company of Kenya Limited (CIC) - Micro health insurance for vendors | Kenya |
| 117 | Kenya Community Based Health Financing Institution (KCBHFA) | Kenya |
| 118 | MobiSure | Kenya |
| 119 | Tanykina Community Healthcare Plan | Kenya |
| 120 | Huduma Poa Health Network | Kenya |
| 121 | Jacaranda Health | Kenya |
| 122 | Assurances Générales du Laos (AGL) | Laos |
| 123 | Netcare Public-Private Partnerships | Lesotho |
| 124 | Adopt-A-Doctor | Liberia |
| 125 | CUMO Microfinance | Malawi |
| 126 | Union Technique de la Mutualité (UTM) | Mali |
| 127 | Universal Health Insurance (Mali) | Mali |
| 128 | L'Union Technique de la Mutualite Malienne | Mali |
| 129 | Djantoli | Mali |
| 130 | Contracting Health Teams and Hospitals in Jalisco, Mexico | Mexico |
| 131 | MAPS Tu Clinica | Mexico |
| 132 | ParaLife | Mexico |
| 133 | Possible Health | Nepal |
| 134 | Dhading and Banke Microinsurance | Nepal |
| 135 | Seguro Facultativo de Salud (Nicaraguan Social Security Institute) | Nicaragua |
| 136 | LAPO | Nigeria |
| 137 | SEAP health foundation | Nigeria |
| 138 | The SureHealth Plan | Nigeria |
| 139 | The River Boat Clinic | Nigeria |
| 140 | Hygeia Community Health Plan (HCHP) | Nigeria |
| 141 | Nigerian Community-Based Health Insurance | Nigeria |
| 142 | Y'ello Health Cover | Nigeria |
| 143 | National Health Insurance System (NHIS) | Nigeria |
| 144 | CAPDAN Microinsurance | Nigeria |
| 145 | Deji Clinic | Nigeria |
| 146 | Pakistan Government of Punjab Contracting-in for Management of Basic Health Units | Pakistan |
| 147 | Punjab Health Foundation | Pakistan |
| 148 | NRSPs Micro-Health Insurance Program | Pakistan |
| 149 | Tameer Sehat O Sukoon | Pakistan |
| 150 | Heartfile Health Equity Financing | Pakistan |
| 151 | Project Hope-Telemedicine Project | Pakistan |
| 152 | Waseela-e-Sehat (Benazir Health Insurance) | Pakistan |
| 153 | Naya Jeevan | Pakistan |
| 154 | RSPN-Adamjee Health Microinsurance Model | Pakistan |
| 155 | ServiPeru | Peru |
| 156 | Club PGN - Loyalty Consumer Plan | Peru |
| 157 | Pro Mujer Peru | Peru |
| 158 | Por Ti, Familia | Peru |
| 159 | PhilHealth Maternity Care Package (MCP) | Philippines |
| 160 | Orange Card Scheme | Philippines |
| 161 | Bicao Investment Care Administration Organization (BICAO) | Philippines |
| 162 | CARD Microinsurance | Philippines |
| 163 | Ipon ni Mommy, Buhay ni Baby (A Buntis Baby Bank Project) | Philippines |
| 164 | BayadLoad | Philippines |
| 165 | Social Health Insurance Indigency Program of Bindoy, Negros Oriental (BSHIIP) | Philippines |
| 166 | Cotabato Health Insurance Program | Philippines |
| 167 | Guimaras Health Insurance Project | Philippines |
| 168 | PhilHealth | Philippines |
| 169 | Provincial Health Indigency Program of Negros Oriental (PHINO) | Philippines |
| 170 | PhilHealth Remittance-By-Air | Philippines |
| 171 | Southern Philippines Medical Center (SPMC) | Philippines |
| 172 | PhilHealth KaSAPI (Kalusugang Sigurado at Abot-Kaya sa PhilHealth Insurance) | Philippines |
| 173 | Provincial Indigency Health Program: The Bukidnon Model | Philippines |
| 174 | Social Insurance of Paracelis, Mountain Province | Philippines |
| 175 | Tarlac Health Maintenance Plan | Philippines |
| 176 | 2nd Women’s Health & Safe Motherhood Program | Philippines |
| 177 | EntrepreNurse | Philippines |
| 178 | Pinoy Health Pass | Philippines |
| 179 | Rwanda Performance-Based Financing | Rwanda |
| 180 | Mutuelles de Sante, Rwanda | Rwanda |
| 181 | Inshuti Mu Buzima | Rwanda |
| 182 | Ocsa care | South Africa |
| 183 | Bloemfontein Hospitals Public-Private Partnership | South Africa |
| 184 | DomestiCare | South Africa |
| 185 | All Lanka Mutual Assurance Organisation (ALMAO) | Sri Lanka |
| 186 | Yasiru | Sri Lanka |
| 187 | Health Services Contracting in Tanzania: Nyakahanga District Designated Hospital | Tanzania |
| 188 | Micro/community health insurance | Tanzania |
| 189 | Tanzania's Community-Based Health Insurance | Tanzania |
| 190 | National Health Insurance Fund (Tanzania) | Tanzania |
| 191 | Tanzania's National Social Security Fund | Tanzania |
| 192 | KNCU Health Plan | Tanzania |
| 193 | Private Nurses and Midwives Association Tanzania (PRINMAT) | Tanzania |
| 194 | Universal Coverage Scheme (Thailand) | Thailand |
| 195 | Sosyal Güvenlik Kurumu (SGK) | Turkey |
| 196 | Faith-Based Not-For-Profit Organization Contracting in Uganda | Uganda |
| 197 | Happy Health Insurance Scheme Clinic | Uganda |
| 198 | Kadic Health Services | Uganda |
| 199 | Munno Mu Bulwadde Microinsurance | Uganda |
| 200 | Charis International Medical Centre | Uganda |
| 201 | Hope Ward at International Hospital Kampala | Uganda |
| 202 | LifeNet International | Uganda |
| 203 | Mother Child Rescue Project (MCRP) | Uganda |
| 204 | Comboni Hospital Health Plan | Uganda |
| 205 | eQuality Health Bwindi | Uganda |
| 206 | International Medical Group | Uganda |
| 207 | Kisiizi Community Health Insurance Scheme (KCHIS) | Uganda |
| 208 | Kitovu Community Health Insurance Scheme | Uganda |
| 209 | Kiwoko Hospital Community-Based Health Insurance | Uganda |
| 210 | Microcare | Uganda |
| 211 | Nyakibale Hospital Health Plan | Uganda |
| 212 | Ishaka Hospital Health Insurance Scheme | Uganda |
| 213 | Kitanga Health Insurance Scheme | Uganda |
| 214 | Mutolere Community Health Insurance Scheme | Uganda |
| 215 | Nyamwegabira Community Based Health Insurance Scheme (NCBHIS) | Uganda |
| 216 | School Health Made Easy | Uganda |
| 217 | Health Care Fund for the Poor (HCFP) | Vietnam |
| 218 | Tinh chi em (Sisterhood) | Vietnam |
| 219 | Da Nang Women’s Hospital | Vietnam |
| 220 | Zambia Universal Health Insurance | Zambia |
